# Pathophysiology of blood brain barrier dysfunction during chronic cerebral hypoperfusion in vascular cognitive impairment

**DOI:** 10.7150/thno.68304

**Published:** 2022-01-16

**Authors:** Vismitha Rajeev, David Y. Fann, Quynh Nhu Dinh, Hyun Ah Kim, T. Michael De Silva, Mitchell K.P. Lai, Christopher Li-Hsian Chen, Grant R. Drummond, Christopher G. Sobey, Thiruma V. Arumugam

**Affiliations:** 1 Memory Aging and Cognition Centre, Department of Pharmacology, Yong Loo Lin School of Medicine, National University of Singapore, Singapore; 2 Department of Biochemistry, Yong Loo Lin School of Medicine, National University of Singapore, Singapore; 3 Healthy Longevity Translational Research Program, Yong Loo Lin School of Medicine, National University of Singapore, Singapore; 4 Centre for Healthy Longevity, National University Health System (NUHS), Singapore; 5 Centre for Cardiovascular Biology and Disease Research, Department of Physiology, Anatomy and Microbiology, School of Life Sciences, La Trobe University, Bundoora, VIC, Australia; 6 School of Pharmacy, Sungkyunkwan University, Suwon, Republic of Korea

## Abstract

The prevalence of cerebrovascular disease increases with age, placing the elderly at a greater lifetime risk for dementia. Vascular cognitive impairment (VCI) encompasses a spectrum of cognitive deficits from mild cognitive impairment to dementia. VCI and its most severe form, vascular dementia (VaD), is becoming a major public health concern worldwide. As growing efforts are being taken to understand VCI and VaD in animal models and humans, the pathogenesis of the disease is being actively explored. It is postulated that chronic cerebral hypoperfusion (CCH) is a major cause of VCI. CCH activates a molecular and cellular injury cascade that leads to breakdown of the blood brain barrier (BBB) and neurodegeneration. The BBB tightly regulates the movement of substances between the blood and the brain, thereby regulating the microenvironment within the brain parenchyma. Here we illustrate how BBB damage is causal in the pathogenesis of VCI through the increased activation of pathways related to excitotoxicity, oxidative stress, inflammation and matrix metalloproteinases that lead to downstream perivascular damage, leukocyte infiltration and white matter changes in the brain. Thus, CCH-induced BBB damage may initiate and contribute to a vicious cycle, resulting in progressive neuropathological changes of VCI in the brain. This review outlines the molecular and cellular mechanisms that govern BBB breakdown during CCH and highlights the clinical evidence in identifying at-risk VCI patients.

## Introduction

Vascular cognitive impairment (VCI) covers an entire spectrum of vascular pathologies that contribute to cognitive impairment, from pre-clinical subjective states to the manifestation of a severe state of cognitive decline such as vascular dementia (VaD) 1,2. Cerebral small vasssel disease (cSVD) is a common age-related condition that drives VCI 3-8. cSVDs are characterised by pathological processes that affect the structural and functional integrity of small arteries, arterioles, capillaries and venules of the brain 9. Some of the distinct clinical features of cSVD observed in VCI patients that can be visualised using neuroimaging include lacunar infarcts, enlargement of the perivascular space, cerebral atrophy, microbleeds, microinfarcts, white matter hyperintensities (WMHs) and reduced cerebral blood flow (CBF) 5,6,8,10-13.

The onset of disease manifestation from risk factors are complicated as risk factors lead to multiple layers of damage before reaching the vascular pathology stage in VCI and VaD. The degree of association between the risk factors and the vascular disease is thought to be dependent on age, lifestyle, genetic susceptibility, comorbidity of other diseases and even differential brain reserves as reviewed in detail previously 2,14,15. Briefly, age has been suggested to be the strongest risk factor for VaD and accounts for many unrecognized vascular changes in the brain. After the age of 65, the risk of developing dementia progressively increases 16. There are studies on sexual dimorphism that argue that the protective effects of estrogen in women against coronary heart disease account for a lower risk of VaD in females 16-19. With regards to lifestyle factors, smoking has been identified as significant risk factors for cardiovascular disease, cerebral vascular disease and cognitive decline. Smoking causes vascular endothelial dysfunction and atherosclerotic damage 20-22. Taken together, these risk factors each contribute to the progression of vascular disease and its unique impact on cognitive impairment. Therefore manipulating some of these risk factors and observing their impact on VCI progression can perhaps provide some pragmatic treatment perspectives to clinicians when identifying vulnerable patients. Understanding the VaD subtypes, lesions and risk factors reflects the complex layers present in people with VaD, and the causes of cognitive impairment are likely to be multifactorial.

Even without the presence of risk factors, vascular aging leads to chronic cerebral hypoperfusion (CCH) that induces phenotypical changes in the brain and therefore makes the brain more vulnerable to disease 23. This emphasizes the importance of CBF regulation under physiological and pathological conditions. Cerebral blood vessels are responsible for the delivery of many important substances to the brain such as nutrients and oxygen, which is necessary for neuronal oxidative metabolism of energy substrates. Neurons have limited capacity for anaerobic metabolism, thus adequate CBF is critically important for function and viability of neurons 24. Cerebral hemodynamics play an important role in maintaining coordinated flow responses in the brain as neuronal tissues require tight coordination between neuronal activity and CBF within the brain parenchyma (known as neurovascular coupling). CBF regulation is particularly well-developed in the brain to ensure coordinated flow responses in the brain 25. The BBB is a relevant component of the microcirculatory response unit during ischemia, as it is involved in hemodynamic regulation of the arterial microcirculatory to allow for increased oxygen extraction from the blood 26. The endothelial cells are involved in producing several vasoactive mediators such as nitric oxide (NO) that have a significant influence on vascular tone thereby influencing CBF 27. However, clinical symptoms of hypoperfusion begin to exhibit when the degree of hypoperfusion is higher than the ability of the cerebral vasculature to autoregulate and allow for the supply of oxygen and nutrients to match the metabolic demand of neurons 26,28.

CCH was reported to be a common feature in all subtypes of VCI 29. In fact, CCH was reported to begin at early stages of VCI and continue till the late demented state of VaD 15,30. Furthermore, in a severe state of VaD, global CBF reduction in patients was reported to be more extensive than age-matched controls and Alzheimer's Disease (AD) patients 31,32. VaD patient cohorts reportedly showed decreased CBF to all parts of the brain 33. Another study reported a 31% decrease in CBF at the frontal cortex and a 39% decrease in CBF at the parietal cortex 34. These studies implemented the use of various CBF measuring techniques on patients to determine any circulation changes, as explained in detail here 24. In brief, there are techniques that are able to measure global CBF changes using invasive intravascular measurements such as nitrous oxide (N_2_O) inhalation and thermodilution. There are many more techniques that are able to measure CBF at a regional and local spatial extent such as positron emission tomography (PET), computed tomography (CT) and dynamic susceptibility contrast magnetic resonance imaging (DSC-MRI), which are all minimally invasive. Other local and regional CBF measurement techniques that are non-invasive and therefore are more popular include arterial spin labelling (ASL), transcranial doppler and thermal diffusion flowmetry (LDF).

Investigations into the pathophysiology of cSVD associated with VCI and VaD revealed endothelial dysfunction and blood-brain barrier (BBB) breakdown in the pathophysiology of cSVD associated with VCI and VaD. In fact, BBB breakdown has been proposed to be a core contributing mechanism in cSVD and associated cognitive decline 35. There is evidence reporting that BBB leakage precedes the development of WMHs, a diagnostic hallmark of cSVD, in a longitudinal study 36. BBB damage typically increases with age in normal-appearing white matter, and is more extensive in the white matter of cSVD patients 37. This suggests that BBB damage leads to cSVD pathology. CCH is often associated with the formation of white matter lesions (WMLs) in the deep structures of the brain 38,39, and accordingly, white matter pathology is commonly observed in clinical cases of VCI.

CCH is also associated with neurodegeneration and varying degrees of cognitive decline within the VCI spectrum 15,29,30,34,40,41. Decreased cerebral perfusion has been reported to be correlated with dementia severity and is also able to predict which individuals with mild cognitive impairment (MCI) will progress to develop a severe state of dementia 42,43. Under CCH conditions, there are many mechanisms that are activated chronically in the brain that cause damage to accumulate, and overcome repair processes. These mechanisms include prolonged energy imbalance, oxidative stress, inflammation, endoplasmic reticulum (ER) stress and mitochondrial dysfunction. These mechanisms drive downstream structural changes such as BBB dysfunction, WMLs, glial activation and cell death activation 35,38,39,44. This suggests that although CCH may not directly cause cognitive deficits, the effect of CCH on cognition is mediated by these mechanistic and structural changes in the brain 10. Several animal models of VCI have been developed on the basis of CCH being the main driver for cognitive decline, and they have been reported to mimic closely the various aspects of pathology relevant to VCI and which are extensively used as a basis to probe pathophysiological mechanisms related to the disease 45.

Combined with a CCH state, vascular risk factors such as hypertension, stroke and hyperlipidemia frequently exist in VCI patients, contributing to BBB damage, all of which can propagate the hypoperfusive state of the brain 46,47. Neuropathological studies have reported an increased BBB permeability in other types of dementia including in AD 48-52; however interestingly, a more extensive BBB permeability was observed among VaD patients compared to AD patients 48. An increase in BBB permeability was observed with increasing WML in the brain of VaD patients 53,54, supporting the pathological link between BBB permeability and white matter pathology. Hypoperfusion-associated BBB dysfunction may be an early feature in the development of WMLs and cognitive decline, and may play a direct pathogenic role in dementia manifestation. This review discusses the physiological state of the BBB while exploring pathophysiological mechanisms that govern BBB breakdown and the resulting effects of BBB damage such as leukocyte infiltration and WML formation.

## The Blood-Brain Barrier in Health and Disease

### Blood-Brain Barrier in a Physiological State

Although the term BBB suggests a rigid structure, it is innately semi-permeable and primarily separates the circulating blood and cerebral parenchymal tissue. The BBB surrounds the microvascular architecture, comprising capillaries, arterioles and venules, throughout most central nervous system (CNS) regions 55. The BBB functions to establish a controlled environment in the cerebral parenchyma by maintaining relatively constant level of hormones, nutrients and water in the brain. This is vital as any fluctuations in the finely-tuned microenvironment of the brain could disrupt CNS homeostasis. The BBB also provides defense against circulating toxins or pathogens that could cause brain infections. Thus, the BBB serves as an important anatomical and biochemical barrier for the CNS. The BBB is comprised of endothelial cells, pericytes, astrocyte end feet and the basement membrane which together, enable these unique structural and functional charactericstics. The close interaction of these cells with the neurons forms the neurovascular unit, which is important in regulating several aspects of brain function, including the regulation of CBF.

#### Endothelial Cells and Tight Junctions

**Figure 1:**
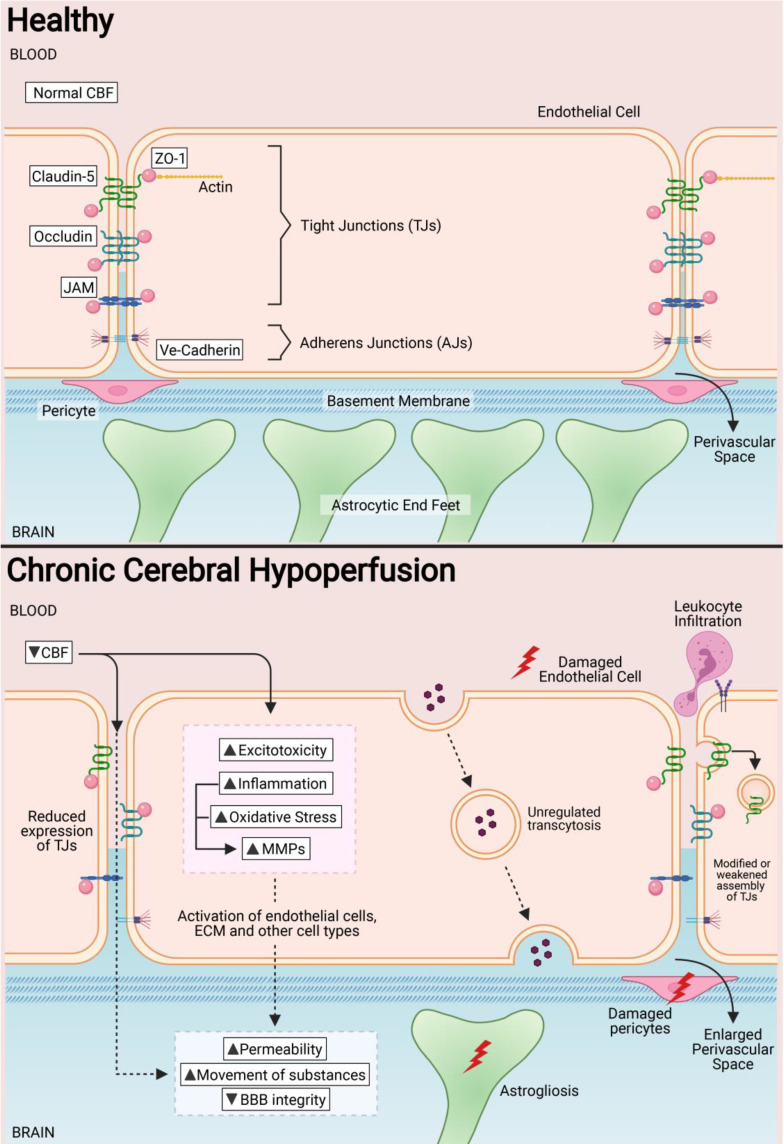
In a healthy state, tight junctions (TJs) zonula occludens (ZO-1), Claudin-5, Occludin and junctional adhesion molecules (JAMs) are involved at the apical end of the endothelial cells. Adherens junctions such as VE-cadherins are involved at the basolateral end of the endothelial cells. Collectively, these proteins provide for a continuous intercellular barrier between the endothelial cells. Pericytes, astrocytic end feet and the basement membrane are associated with the endothelial cells that collectively form the blood brain barrier (BBB) and are involved in the tightly regulated movements of molecules between the blood and the brain. In a chronic cerebral hypoperfused state, the reduced cerebral blood flow (CBF) triggers a myriad of effects on the BBB such as the reduced expression, modification and weakened assembly of the TJ proteins between the endothelial cells. Other effects of a reduced CBF are the induction of increased excitotoxicity, inflammation, oxidative stress and expression of matrix metalloproteinases. Collectively, these effects increase the permeability of the BBB at the endothelial cells and therefore increase the movement of substances between the blood and the brain. Secondary effects of increased BBB permeability include leukocyte infiltration, unregulated transcytosis, damaged pericytes, increased perivascular space and astrogliosis.

Endothelial cells are the primary components of the BBB, responsible for the physical barrier between the circulation and the CNS. Compared with peripheral endothelial cell, CNS endothelial cells have specialised characteristics such as the lack of fenestrations and minimal transcytosis activity that greatly restricts vesicle-mediated transcellular movement of solutes. These cells also have an abundant mitochondrial content, which is important for generation of adenoside triphosphate (ATP) during the transport of substances through ATP-dependant pumps 56. Compared to peripheral endothelial cells, CNS endothelial cells express low levels of leukocyte adhesion molecules, restricting immune cell adhesion and infiltration into the brain parenchyma. Paracellular transport is also restricted by tight junctions (TJs) at the apical end of endothelial cells 57. TJs are unique to brain endothelial cells and provide a “sealing” capacity for the BBB. The structural and functional properties of TJs are attributed to several proteins that interact and form complexes at the BBB, namely the cytoplasmic zonula occludens (ZO) and the transmembrane proteins (i.e. claudins, occludins, and junctional adhesion molecules (JAMs)) 58. ZO proteins are scaffold proteins that directly interact with other proteins in the TJs, and maintain their expression at the transendothelial interface of the BBB and in signal transduction and transcriptional modifications 59,60. Claudin subtypes 1, 3, 5 and 12 are expressed at endothelial TJs, but claudin 5, in particular, makes up a major portion of the BBB 61. Claudins are involved in the tightening of paracellular junctions between endothelial cells and in the maturation of the BBB 62. Occludins are the most abundant protein at TJs, and is important in maintaining the transendothelial electrical resistance at the BBB, and in the assembly of Claudin-5 proteins at the TJ interface 63. In addition to TJs, adherens junctions (AJs) are associated with TJs to mediate their assembly and maintenance. AJs are located on the basolateral end of endothelial cells, and are primarily involved in adhesion of endothelial cells to one another 64. Vascular endothelial cadherins (VE-cadherins), neural cadherins (N-cadherins) and epithelial cadherins (E-cadherins) are the AJs present in brain endothelial cells, with VE-cadherins the most abundant 65 (**Figure 1**).

#### Other BBB Components

Pericytes are involved in the regulation of BBB homeostasis by maintaining low levels of transcytosis at endothelial cells 66. Pericytes are also important within the pericyte-endothelial interactions for modulating CBF within capillaries 67, and in maintaining the structural integrity of the BBB as they modulate the basement membrane and TJ structure and function 68. In addition, pericytes are involved in fine-tuning inflammatory responses in the brain by controlling leukocyte infiltration 69. The position of pericytes around endothelial cells restricts the migration of leukocytes into brain parenchymal tissue. Leukocyte infiltration increases when there is pericyte gap enlargement, increased expression of adhesion molecules such as intercellular adhesion molecule-1 (ICAM-1) and secretion of migration inhibitory factor (MIF) in response to inflammatory signals 70,71. Astrocytes are also essential for the maintence of the BBB. Mature astrocytes are able to extend their end feet processes to completely ensheath the brain microvasculature. These astrocytic end feet processes also extend out to the neuronal circuity, making astrocytes a central link between the microvasculature and neuronal network. Astrocytes can sense the metabolic demands of neurons, and relay signals to the vasculature to coordinate CBF changes to a focal brain area 72. Moreover, astrocytes function to provide barrier properties onto endothelial cells in the brain 73. The vascular basement membrane surrounding the BBB is a component of the extracellular matrix produced by mature endothelial cells 74. Common constituents of the basement membrane include specific laminin isoforms, type IV collagens and proteoglycans. The basement membrane and its protein components provide structural support to anchor the components of the BBB tightly together, while allowing for interactions between various cell types during signal processing 75. In a mouse model of multiple sclerosis, deletion of the laminin α2 within the vascular basement membrane results in increased infiltration of T lymphocytes into the brain 76. Another study reported haemorrhage in deep brain regions and impaired vascular smooth muscle cell function following deletion of astrocytic laminin α2 77. These studies validate that the composition of the vascular basement membrane is important in the integrity and function of the BBB.

### BBB in Chronic Cerebral Hypoperfusion (CCH)

Pathological states in the CNS have been associated with BBB dysfunction. The BBB is intrinsically dynamic, hence it has important functional implications during development, the physiological aging process and age-associated neurodegenerative diseases and cognitive performance. Therefore, it is unsurprising that increased BBB permeability is observed in VaD patients 10,52,78-83. An increased BBB permeability allows for uncontrolled movement of substances between the blood and brain parenchyma, which may include entry of immune cells and pathogens into the brain. Impairment of the BBB can be further aggravated when endothelial cells and other components of the neurovascular unit, such as pericytes, astrocytes and basement membranes, are affected. During CCH, the anatomical and functional properties of TJs at the endothelial cell interface can be modulated by changes in the expression of TJ proteins during hypoperfusion. In fact, increased BBB permeability in VaD has been reported to occur with neuronal loss and white matter degeneration during disease progression 84. BBB damage generally propagates through various molecular cascades of events due to CCH. These mechanisms include, but are not limited to excitotoxicity, oxidative stress, inflammation and activation of matrix metalloproteinases (MMPs). Concurrently, BBB damage induces a series of downstream events that contribute to secondary brain injury, such as perivascular damage, astrogliosis, leukocyte infiltration and WML formation (**Figure 1**).

### Primary Pathological Mechanisms Underlying BBB Damage During CCH

#### Excitotoxicity

CCH induces cellular energy imbalance and consequently, a state of excitotoxicity in the CNS 75,85-87. Excitotoxicity is defined as the state of increased activation of neurons in response to increased levels of extracellular glutamate and subsequent calcium ion (Ca^2+^) influx 88. High levels of glutamate release during excitotoxicity cause a persistent activation of N-methyl-d-aspartate acid (NMDA) receptors, ∝-amino-3-hydroxy-5-methylisoxazole propionic acid (AMPA) receptors, and metabotropic glutamate receptors (mGluRs) on neurons during CCH. Consistent with neuronal excitotoxicity, the released glutamate activates glutamate receptors on other brain cells such as endothelial cells, astrocytes and microglia.

**Figure 2:**
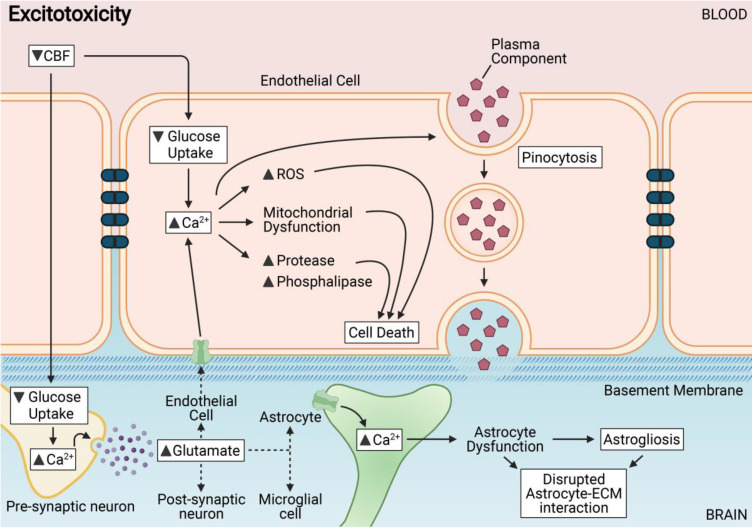
Chronic cerebral hypoperfusion or reduced cerebral blood flow (CBF) results in various mechanisms triggered in endothelial and neighbouring cells of the neurovascular unit including excitotoxicity. Excitotoxicity is induced in endothelial cells in response to a decreased glucose intake. Abnormally high levels of cytosolic Ca^2+^ induces the disruption of metabolism, mitochondrial dysfunction, activation of proteases and phospholipases, and production of reactive oxidative species (ROS) that collectively contribute to cell membrane damage and vascular cell death leading to disruption of BBB integrity. Astrocytes of the BBB are adversely affected by excitotoxins, as the interaction between astrocytes and the extracellular matrix (ECM) is rapidly disrupted. Astrocytic dysfunction or astrogliosis coupled with increased transendothelial pinocytosis contributes mostly to BBB breakdown after excitotoxicity.

Endothelial cells express NMDA receptors, and activation of these receptors during excitotoxicity results in calcium signaling and downstream NO production that promotes vascular permeability in the brain 89-91 (**Figure 2**). Persistent activation of glutamate receptors at the BBB during CCH may promote increased movement of Ca^2+^ into the endothelial cytosol, which induces disruption of metabolism, mitochondrial dysfunction, activation of proteases and phospholipases, and production of free radicals that can collectively contribute to membrane damage, vascular cell death and disruption of BBB integrity 92,93. Another consequence of excitotoxicity is the modulation of ion channels and transporters at the BBB. Ion exchangers that carry ions such as sodium, potassium, chloride and calcium, and membrane transporters such as ATP-binding cassette (ABC) transporters are responsible for the maintenance of physiological and metabolic homeostasis, as well as ionic balance at the BBB. Increased vesicular transport across endothelial cells within a leaky BBB contributes to vasogenic edema. High Ca^2+^ levels in the cytosol can also lead to increased phosphorylation and activation of myosin light chain kinase (MLCK) 94. Activated MLCK leads to the contraction of actin in the cytosol and relocation of TJ proteins between endothelial cells 95.

Astrocytes of the BBB have been shown to be adversely affected by persistent and excessive glutamate release, as astrocyte interaction with the extracellular matrix is rapidly disrupted 96. Endothelial cells have been reported to in fact be highly resistant to primary glutaminergic stimuli and prevent the passive transport of glutamate into the brain parenchyma 97. Glutamate has, interestingly, no direct effect on the expression of TJ proteins between endothelial cells, but is involved in the trigger of pinocytosis - a transendothelial transport process 98. Thus, astrocytic dysfunction coupled with increased transendothelial pinocytosis contributes mostly to BBB breakdown during excitotoxicity.

Microglia are resident immune cells that play an essential function in immune surveillance, host defense against injury and tissue repair in the brain. CCH-induced excitotoxic stimuli have been reported to alter microglial function and can contribute to the pathogenesis of chronic CNS diseases 99. Upon excessive and persistent glutamate release, activated microglia initiate several responses that elevate signaling pathways related to neurotransmitter homeostasis, ATP energy utilization and macromolecular synthesis in the brain 100. In light of the importance of microglia in the brain, dysregulation of microglia activity may severely hamper its capacity for combating inflammatory challenges and tissue repair in the brain 101. Overactivated microglia can contribute to neurotoxicity by becoming a source for glutamate release in the brain further modulating the pathogenesis of BBB disruption during CCH.

Interest in targeting excitotoxicity expanded following its implication in the pathogenesis of ischemic brain injury but there are little evidence in clinical trials that prove its efficacy 102. However, there has been a gain of interest in this field in recent years that suggests that this knowledge should be leveraged to develop neuroprotective treatment against VaD. Glutamate antagonists against hypoperfusion have been reported to fail in clinical testing 103,104. It is also noted that pan blockade of NMDA receptors could lead to anti-excitotoxic efficacy but widespread loss of fast synaptic excitation would not be well tolerated, therefore admistration of these drugs at the correct time is cruicial and may bring back these drugs into active consideration in clinical trials 102. A combination approach of other drugs with anti-excitotoxic efficacy may be a more favourable approach.

#### Oxidative Stress

Hypoxia leads to increased production of reactive oxygen species (ROS) and reactive nitrogen species (RNS) in the brain microvascular endothelium, thus leading to oxidative stress 105. Mechanistically, hypoxia leads to a coordinated downregulation of metabolic utilization of ATP and ultimately a bioenergetic collapse. The cellular production of ATP is usually due to a decrease in activity of the electron transport chain in the mitochondria 106. Complex IV is the main enzyme in the electron transport chain (ETC) that utilizes oxygen and couples it to the generation of ATP. During low oxygen levels, the electron transport chain is the major site for ROS production 107. The three main sources of oxidative stress in the vasculature are the nicotinamide adenine dinucleotide phosphate (NADPH) oxidases, mitochondria and cyclooxygenases (COX) 108. While there are several ROS species such as peroxides, superoxides, hydroxyl radicals, many studies have suggested that superoxide plays a key role in BBB damage 109-112. During CNS injury, the cellular defense systems are weakened, and ROS species will lead to the oxidation of lipids, proteins, and nucleic acids that alter endothelial cell function, therefore leading to BBB dysfunction 113. Patients with VaD have reported increased levels of malondialdehyde, a marker of lipid peroxidation, as compared to controls, and even higher than those reported in AD patients 114.

**Figure 3:**
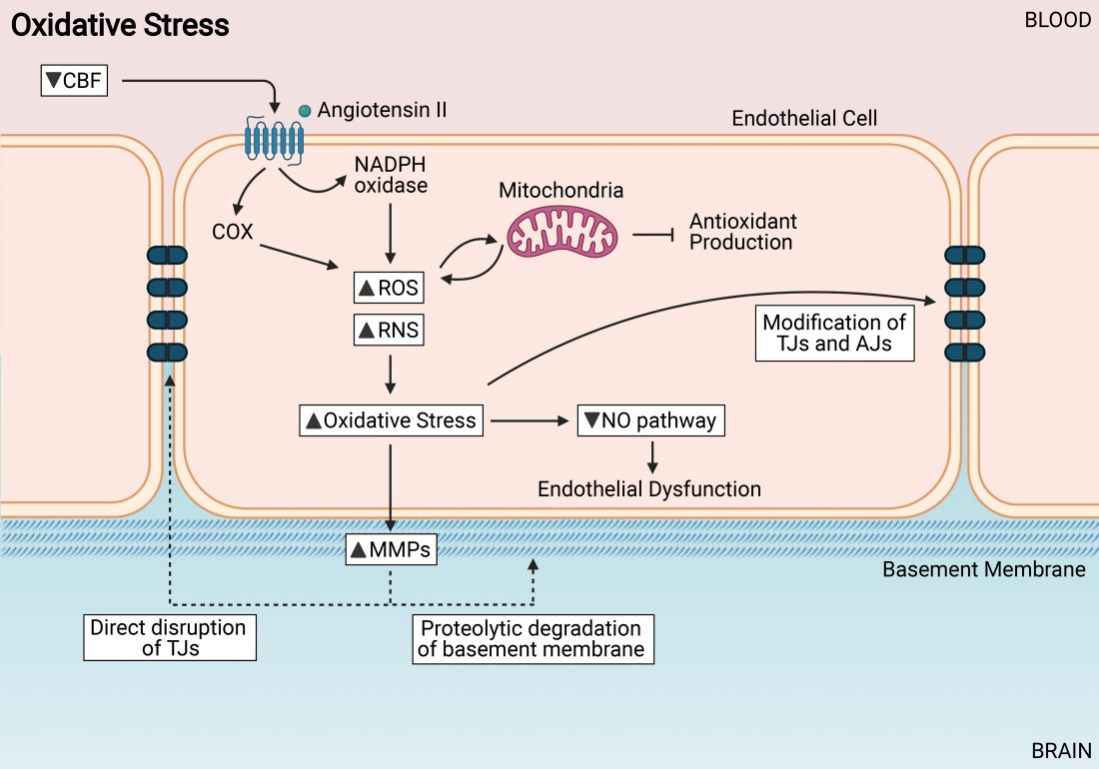
Oxidative Stress induces endothelial cell dysfunction. Increased reactive oxygen species (ROS) and reactive nitrogen species (RNS) from sources such as NADPH oxidases, cyclooxygenases and mitochondria. Increased oxidative stress can modify TJs directly, cause endothelial dysfunction, and activate MMPs.

NADPH oxidases have been reported to be involved in the progression of VaD states 115-117. Angiotensin II is an important inducer of NADPH oxidase-modulated superoxide production and endothelial dysfunction within the BBB 118. Angiotensin II has also been implicated in cognitive impairment and dementia 119. Mitochondria are abundant within endothelial cells and these organelles are activated by NADPH oxidases to generate ROS. COX was shown to increase ROS production in endothelial cells through pro-inflammatory responses 120. ROS may affect BBB integrity by modifying the interface of TJ proteins and inducing a decrease in E-cadherin levels, which are important for BBB maintenance 121 (**Figure 3**).

An increase in ROS in the endothelial cells leads to an impairment of NO signaling 122. In physiological conditions, NO is a vasodilator controlling CBF; it is also a neurotransmitter that regulates vascular smooth muscle cells; it regulates Ca^2+^ levels in the brain therefore protecting neurons from excitotoxic damage; and is important in preserving and maintaining learning and memory function 123. NO bioavailibility is decreased in endothelial cells during CCH as it can react with superoxide (O_2-_) to form peroxynitrite (OONO^-^), causing extensive oxidative stress, endothelial dysfunction and gradual BBB impairment 120 (**Figure 3**). Another pathway that increases oxidative stress in the BBB is through the upregulation of the receptor for advanced glycation end products (RAGE). Upregulation of RAGE results in the activation of downstream nuclear factor kappa beta (NF-κB) pathways which eventually lead to the dysregulation of the BBB via reduction in the TJ proteins between endothelial cells and thus increasing BBB permeability 124-126. This pathway might play an important role in the pathogenesis of BBB breakdown during CCH.

Given the association between increased oxidative stress during CCH and increased BBB permeability, preventing or inherently reversing BBB breakdown through antioxidant therapies may be a target for the treatment of VaD as described here 127. Antioxidant therapy such as ocimum sanctum (OS) has been reported to reduce lipid peroxidation after CCH was induced in rats 128. Another popular antioxidant treatment is resveratrol, which reported a reduced lipid peroxidation and restored glutathione levels in rat brains following CCH 129. Scavengers of ROS such as edaravone have been reported to ameliorate oxidative stress under CCH and also rescue white matter integrity and memory function in rats 130. Another antioxidant therapy Chotosan enhanced antioxidative pathways in rats subjected to CCH, reversing ROS production and ameliorate memory impairment 131. There are no trials for antioxidant therapies in VaD to date, although there are completed and ongoing clinical trials for AD 132. There may therefore be potential in repurposing some of the antioxidant therapies that have had clinical success into VaD patients to examine their effects.

#### Inflammation

Although the brain has historically been considered to be an immune-privileged site, it is now understood that the CNS and the immune system are well connected and inflammation is involved in vascular and tissue remodelling after injury. With the reduction of CBF, an ischemic cascade consisting of a complex series of events is initiated that ultimately lead to activation of various brain cells to induce their death. The ischemic cascade triggers expression of pro-inflammatory cytokines, chemokines, and inflammatory mediators by various brain cells. Excitotoxicity, oxidative stress, microvascular injury, BBB dysfunction and secondary inflammation can exacerbate hypoperfusion injury in the brain and lead to permanent cerebral damage. In VaD, inflammatory genes and pathways are upregulated during disease progression 133, and so there has been increased interest in translational research to mitigate brain injury following CCH.

The cerebral vasculature is an important site of neuroinflammation and the BBB becomes compromised under inflammatory conditions 134. Inflammation can affect the primary component of the BBB - the endothelial cells - through a number of different mechanisms. These include formation of membrane abnormalities that can compromise the integrity of the BBB via increased fenestrations; increased damage to the mitochondria that can prevent regulated transcytotic diffusion across the BBB and activation of apoptotic signals that can lead to endothelial cell death 135. Generally, pro-inflammatory cytokines and chemokines activate specific signalling cascades that increase BBB permeability at the endothelial cell interface through weakening of the assembly and expression levels of TJ proteins 136-142. These pro-inflammatory cytokines also increase expression of cell adhesion molecules on the endothelial cell surface of the BBB which increases leukocyte infiltration 143-150. It was also shown that CCH-induced pro-inflammatory cytokines can activate Rho-kinase signalling within endothelial cells 151. The Rho-kinase pathway is involved in the internalisation of TJ proteins via endocytosis that can directly influence the disruption and permeability of the BBB 152. During chronic inflammatory states such as in VaD, the internalised TJ proteins may not be recycled to the plasma membrane, but are instead directed to the lysosome for degradation, leading to permanent BBB dysfunction 153.

**Figure 4:**
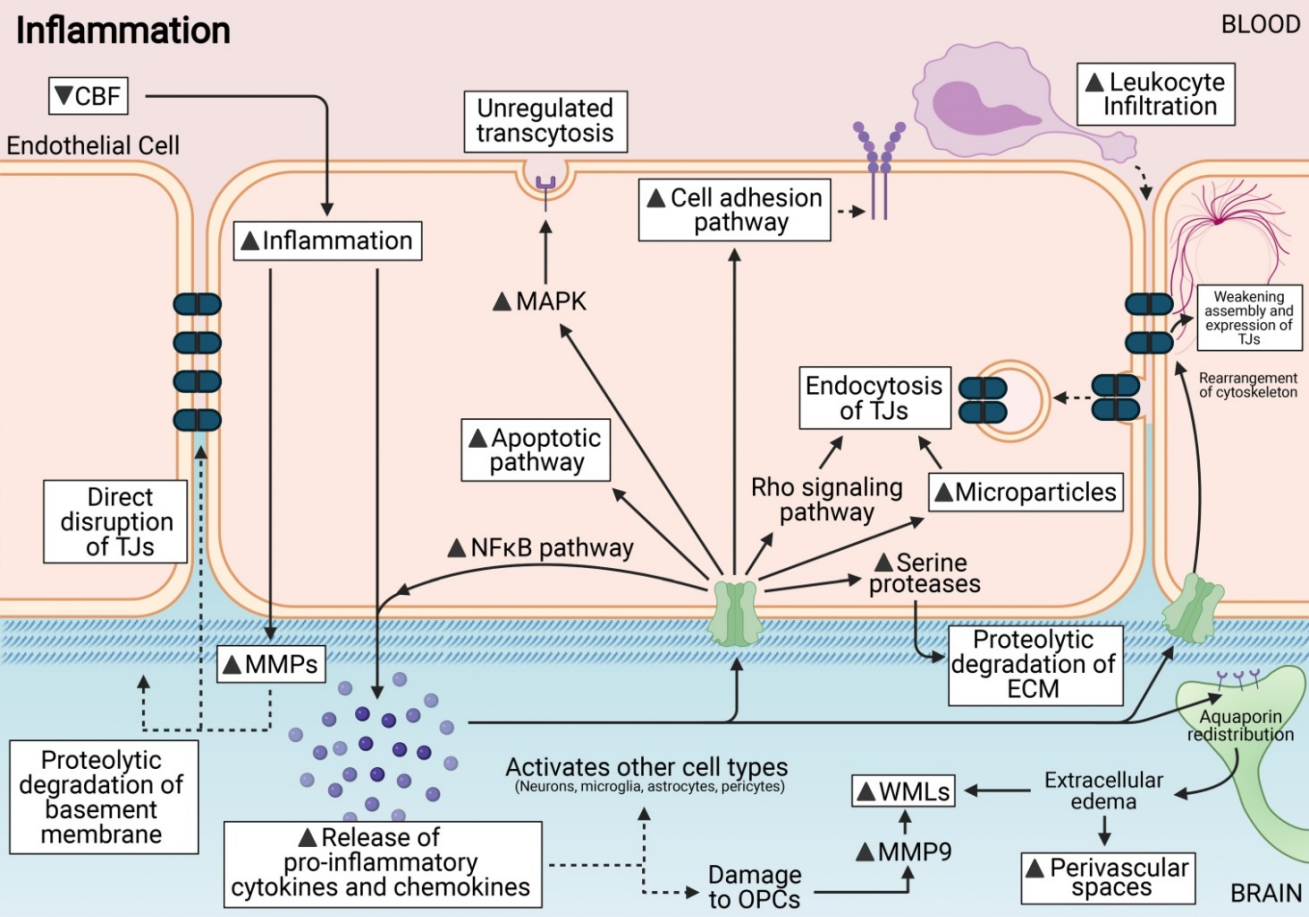
Inflammation is induced via the release of pro-inflammatory cytokines and chemokines. This release can activate various cell types including endothelial cells. These cytokines can directly damage the BBB, and increase permeability at the endothelial cell interface via either weakening assembly or endocytosis of tight junctions (TJs). They can also activate apoptotic pathways; unregulated transcytosis across the endothelial cells and also occur in response to inflammation. MMPs are activated by inflammation and can affect ECM and TJ disruption. Leukocyte infiltration is a downstream effect of increased cell adhesion pathways during inflammation. ECM edema can also cause other downstream effects such as white matter lesions (WMLs) and perivascular damage.

In addition, microglia plays a central role in orchestrating neuroinflammation in the brain. Activated microglia have a pro-inflammatory phenotype that contributes to BBB damage by activating downstream inflammatory mediators such as interleukin-1 beta (IL-1β), tumour necrosis factor (TNF), interleukin-6 (IL-6), and monocyte chemoattractant protein-1 (MCP-1) 154,155. These cytokines released by activated microglial cells can directly and indirectly contribute to BBB damage 156,157. In particular, it was reported that necroptosis, a regulated mode of inflammatory cell death, was the underlying mechanism responsible for endothelial cell death contributing to BBB disruption 154 (**Figure 4**).

Here we summarize various cytokines and chemokines formed during CCH and discuss the role of each in BBB breakdown. Inflammatory cytokines such as IL-1β, IL-6, interleukin-17 (IL-17), interferon gamma (IFN-γ), TNF and C-C motif Chemokine Ligand 2 (CCL2) have been reported to be increased during CCH 158-161. This suggests that CCH-induced inflammatory responses in various cell types may play an important role in neurovascular pathology.

##### Interleukin-1β (IL-1β)

In response to CNS injury, IL-1β expression has been reported to be elevated in various CNS diseases. In humans, IL-1β was elevated in the plasma of VaD human patients and in the cortex, hippocampus, striatum and microglial lysates in mouse models of VaD 158,162,163. IL-1β can be produced by various cell types including microglia, astrocytes, infiltrating leukocytes, neurons and oligodendrocytes, and its primary effects are exerted mainly on microglia, astrocytes and endothelial cells, all of which contribute to BBB integrity 164,165. IL-1β induces strong inflammatory effects on endothelial cells, which induces BBB leakiness via reduced expression of TJ protein ZO-1 166. In particular, IL-1β is produced by the inflammasome - a caspase-1 activating molecular platform expressed in various cell types 163. Activation of caspase-1 in the brain increased adhesion and transmigration of peripheral immune cells across the BBB 167. Increased inflammasome-mediated pyroptosis in damaged endothelial cells also contributes to BBB insult 168. Additionally, IL-1β has a positive feedback mechanism where it can trigger an increase in its own expression in the brain 169, thus promoting increased leukocyte infiltration and exacerbating the inflammatory response in the brain 166.

##### Interleukin-6 (IL-6)

IL-6 has both pro-inflammatory and anti-inflammatory properties. Increased levels of IL-6 have been reported in the cerebrospinal fluid of VaD patients when compared to AD or cerebrovascular disease patients 159. It has been reported that high levels of IL-6 in the brain reduces occludin and claudin-5 expression between the endothelial cells 121,137. Therefore, this reduction in TJ proteins can weaken BBB integrity during CCH.

##### Interleukin-17 (IL-17)

IL-17 is a pro-inflammatory cytokine secreted by T cells to initiate neutrophil recruitment. IL-17 primarily exerts neuroinflammatory properties, but also promotes oxidative stress by inducing NADPH oxidase 170. IL-17 is also a potent mediator of secondary chemokine release, activation of endothelial cells, and recruitment of other immune cells 171. Consistent with these findings, in a mouse model of ischemic stroke, IL-17 exacerbated inflammatory responses following ischemia, increasing infarct size and worsening neurological outcomes 172. Serum IL-17 was reported to be higher in VaD patients than in control patients 173. Specifically, at the BBB, IL-17 has been reported to decrease occludin and claudin 5 expression between endothelial cells, leading to BBB leakage 174. Thus, increased IL-17 in VaD brains is likely to also reduce expression of TJ proteins and BBB integrity 173.

##### Tumour Necrosis Factor (TNF)

TNF is a multifunctional cytokine secreted by several cell types including macrophages, natural killer cells, and lymphocytes, and plays a key role in the initiation and perpetuation of cerebral inflammation. Activation of TNF production has been implicated in various diseases including VaD and AD 143,175. Exogenous TNF administration increased BBB permeability in control mice and in ischemic states 176. At the BBB, inceased TNF expression in endothelial cells result in a rearrangement of the cytoskeleton within the cytoplasm, thus disrupting TJ protein assembly and basement membrane 177-180. TNF also activates NF-κB and mitogen-activated protein kinase (MAPK) signaling pathways, whose major targets include the increased transcription of other pro-inflammatory cytokines and apoptosis related genes, all contributing to the activation and dysfuntion of endothelial cells 181-184. TNF upregulates expression of toll-like receptors (TLR2, TLR3, TLR4 and TLR6) in various cell types including endothelial cells. In particular, activation of TLR2 at endothelial cells increases BBB permeability via disruption of TJ proteins occludin and claudin-5 185. Peripheral levels of TNF are reported to be involved in BBB disruption at the endothelial interface as increased serum TNF levels downregulate occludin expression, thereby inducing BBB disruption in a mouse model of liver failure 138. Peripheral TNF levels have been reported to be increased in VaD patients compared to control subjects 186,187.

##### Interferon gamma (IFN-γ)

IFN-γ is a cytokine that plays an important role in both innate and adaptive immunity. It is secreted by activated T cells and natural killer cells and functions as a primary activator of macrophages. TNF and IFN-γ are cytokines that act synergistically in response to inflammation during pathology. BBB endothelial cells express TNF and IFN-γ receptors, and TNF itself has been reported to independently increase IFN-γ receptors on endothelial cells 188. These cytokines bind to their receptors on endothelial cells to induce various signaling cascades including the recruitment of extracellular signal-regulated kinases (Erk1/2) 188. TNF and IFN-γ synergistically increase chemokine secretion in CNS inflammation 189, and also stimulate the release of microparticles, cell membrane-derived particles generated by budding from the endothelial cell surface 190. These microparticles export TJs and AJs away from the transendothelial surface following cytokine stimulation, thus contributing to the loss of the barrier property of the BBB 190.

##### Chemokine Ligand 2 (CCL2)

Chemokine CCL2 is the main effector of monocyte transmigration during CNS inflammation and is associated with endothelial dysfunction 191. CCL2 participates in increasing BBB permeability during monocyte infiltration into the brain parenchyma 192. CCL2 contributes to BBB opening by the dysregulation of the AJs between the endothelial cells 140. AJs play a role in maintaining mechanical strength and stability of endothelial cells, thus with increased CCL2 expression their dysregulation can lead to increased BBB permeability. Consistent with this, in older adults, CCL2 is reported to be increased in the brain, and is associated with longitudinal decline in memory 193.

Based on many lines of evidence as highlighted above, inflammation is a major pathophysiological mechanism in BBB injury. This evidence highlights the potential importance of looking at anti-inflammatory therapies to combat CCH associated BBB injury. The use of non-steroidal-anti-inflammatory drugs (NSAID) and statins have been reported to reduce BBB damage through suppressing inflammation, particularly within the hippocampus 194. Morin, a bioflavonoid, had been reported to attenuate BBB disruption after cerebral hypoperfusion via its anti-inflammatory properties 195. Glucocorticoid steroids have been reported to increase the tightness of the BBB 196. These steroids influence TJ protein expression between endothelial cells, transendothelial electrical resistance and ZO-1 expression 197. A major limitation with employing steroids as a potential therapeutic intervention is determining the concentration, and the form of the steroids administered, as administration is highly dependent on age, gender, health, diet and the source of the steroids 198. However, to date, there are no novel treatment agents with disease-modifying activity against VaD. There have, however, been many observational studies that indicate the potential of NSAIDs in limiting the progression of dementia as a whole 199.

#### Matrix Metalloproteinases (MMPs)

MMPs are zinc-dependent enzymes responsible for the proteolytic degradation of the extracellular matrix in the brain. In pathological states such as hypoxia and CCH, MMPs are activated by oxidative stress and inflammation in endothelial cells 153,200. MMP substrates include fibronectin and laminins within the basal lamina, which provide a structural scaffold for endothelial cells in the BBB 75. Although the basement membrane is the main substrate of MMPs, TJ proteins are also MMP substrates. Occludin, claudin-5 and ZO-1 proteins are degraded by MMP2 and MMP9 during CCH 153,201,202. Thus, MMPs are robustly and directly involved in disruption of BBB structural integrity and hence its permeability. Activated microglia are the main source of MMP activation in the brain during CCH. In the hypoxic state of VaD, microglial MMP2 and MMP9 are increased 203, and are involved in the degradation of the basement membrane and TJ proteins of the BBB 204. Though MMP2 and MMP9 enzymes are both upregulated during CCH, MMP2 expression in the microglia and vascular endothelium of white matter contributes more extensively to the remodeling of white matter myelin 205.

Upstream of the MMP pathway is the activator known as cyclophilin A (CypA). The CypA protein has multiple functions including protein folding, protein trafficking, assembly and cell signalling, and is activated by oxidative stress and inflammation 206. CypA is activated in various human diseases including dementia, and the primary mechanism through which it exerts its inflammatory effects is through inducing an imbalance in the MMP levels in the brain and altering collagen turnover and extracellular remodelling, thus affecting vascular structure 207-209. Changes in cell-matrix attachment sites (integrins) and their topographical localization may modulate arterial structure. Specifically at the BBB, both intracellular and extracellular CypA contribute to inflammation by promoting expression of adhesion molecules and enhancing T cell responses 210.

In this context, recent years have witnessed tremendous advancements in the field of MMP research, with a special emphasis for neuroprotection at the site of the BBB. Since MMPs have been implicated in the pathology of BBB insult, there has been much interest in the use of MMP inhibitors to treat diseases, and hence improve BBB integrity. Over the past decade, there has been better understanding of MMP activity and its influence on the BBB, and specific unique MMP inhibitors have been designed. Until recently, these MMP inhibitors have not been used in clinical trials, as MMP inhibitors have been designed to have desirable selectivity and improved pharmacokinetics 211. However, the reluctance of using MMP inhibitors still exists as the same MMP is able to exert both beneficial and non-beneficial roles, and also have differing roles in different organ systems 212.

### Pathological Secondary Effects Underlying BBB Damage During CCH

#### Perivascular Damage

A consequence of BBB damage is an increased size of perivascular spaces, a common observation in VaD. Perivascular spaces are part of an intricate fluid clearance system in the brain, and its dysfunction in disease results in accumulation of harmful waste products in the brain 213. BBB damage includes the damage of pericytes. Although a direct link of pericytes to VaD is not clear, studies have shown that pericyte damage is associated with CCH, white matter damage, neuronal loss and cognitive impairment 214.

#### Leukocyte Infiltration

The absence of leukocytes in a healthy brain is due to restriction of leukocyte infiltration into the CNS by the BBB 215. However, loss in BBB integrity during CCH can lead to uncontrolled movement of substances, resulting in glial activation and an altered extracellular environment around neurons. During CCH, a hypoxia-mediated inflammatory response triggers leukocyte infiltration through increased expression of pro-inflammatory chemokines and cytokines. Recruitment of these inflammatory cells into the microvasculature further compromises vessel perfusion, contributing to a persistent state of neuroinflammation in CCH 216.

Activation of endothelial cells in response to inflammation initiates a coordinated and sequential recruitment of inflammatory cells into the cerebral vasculature, including populations of neutrophils and mononuclear leukocytes such as lymphocytes, monocytes, macrophages and dendritic cells, that have been extensively described 216,217. Briefly, adhesion molecules such as ICAM, vascular adhesion molecule (VCAM), selectins and leukocyte integrins are commonly expressed on endothelial cells to aid leukocyte infiltration. Selectins are involved in the tethering and rolling interactions of endothelial and leukocyte recruitment by binding to fucosylated oligosaccharide ligands on circulating leukocytes 218. ICAMs and VCAMs allow for the arrest of leukocytes, and a subsequently tighter adhesion of leukocytes onto the endothelial cells 219. Integrins expressed on leukocytes allow for the transmigration between endothelial cells into parenchymal tissue 220. Therefore, increased expression of adhesion molecules on endothelial cells collectively accelerates leukocyte infiltration into the brain 216.

At endothelial cells, increased expression of chemokines, cytokines and P-selectin collectively activates the NF-κB pathway to mediate increased expression and recruitment of adhesion molecules on cell surface membranes 221,222. TNF expression decreases BBB integrity via upregulation of ICAM and selectins on endothelial cells 223. Activated leukocytes contribute to a chronic inflammatory state of CCH through further release of pro-inflammatory cytokines, forming a positive feedback loop of endothelial activation that perpetuates BBB damage. Leukocytes recruited into the brain also produce ROS, which exacerbates brain injury from immune cell infiltration by activating the NF-κB pathway.

Pericytes also play a role in leukocyte infiltration into the brain in CCH-mediated inflammation. Pericytes coordinate the navigation of infiltrated cells and regulate the type of cells infiltrating the brain by controlling the opening size of intra-pericyte gaps 70,71,224,225. This controlled relaxation and opening of pericytic gaps is coordinated by Rho and its downstream Rho-assocated forming protein kinase (RhoA/ROCK) signaling pathway 225. In addition, the basement membrane has also been recently reported to mediate leukocyte extravasation into the brain 76; it was reported that there was a limited ability for T cells to enter the CNS due to the presence of laminin 5 expressed on endothelial cells, therefore potentially reducing disease susceptibility and severity.

Endothelial inflammation is associated with excessive activated platelets. Platelets are part of the body's defence system when there is excessive bleeding. The presences of activated platelets in circulation induce leukocyte adhesion on activated endothelial cells and promote inflammation in the brain 221,226,227. This is followed by increased transcription and expression of adhesion receptors that mediate the docking and extravasation of leukocytes into the brain parenchyma under pathological conditions 228. In VCI, vascular abnormalities such as disrupted microvascular integrity and microbleeds may trigger platelet activation 229. The uncontrolled activation of platelets in VaD can result in chronic inflammatory reactions that can mediate endothelial cell stress. Besides having a key role in inflammation, platelets play an important role in hemostasis, whereby they initiate clotting following endothelial disruption or tissue injury. Platelets release substances such as thromboxane A2 that can deposit in the walls of cerebral blood vessels, which ultimately translates into vascular degeneration 230. Platelets have gained much interest in recent years as they respond rapidly to environmental changes and potentially be a valid cellular tool to study pathogenesis of VaD.

In summary, increased BBB permeability allows infiltration of peripheral leukocytes. Activated leukocytes can further increase BBB permeability by exacerbating the inflammatory response via disruption of TJ proteins between the endothelial cells, modifying basement membrane proteins, activating pericytes and platelets. Yet, in contrast to their involvement in BBB damage, extravasation of immune cells in the brain increases migration of protective anti-inflammatory cells into the brain that may contribute to the repair of the BBB 231. This emphasizes the need to identify the precise molecular and cellular pathways involved in BBB-induced leukocyte infiltration into the brain during CCH.

### White Matter Damage

WMLs are associated with cognitive dysfunction in VaD patients and involve injury to oligodendrocytes. The pathogenesis of WMLs is associated with ischemic conditions in the brain 232-234. In fact, many studies emphasize the role of damaged BBB in the induction and progression of WMLs 235-237. A rodent model of CCH underlines the importance of an intact BBB in WML formation as it suggests that BBB damage is the starting point for WML formation 47. Following white matter injury, oligodendrocyte precursor cells (OPC) proliferate and differentiate into mature cells in an attempt to restore damaged white matter. However, the proliferation capacity of OPCs is limited as CCH-induced pro-inflammatory cytokines disrupt the ability of vascular endothelial cells to support OPCs 238. OPCs rapidly respond to early white matter damage and express MMP-9 endopeptidases, which further aggravates BBB disruption 239. A higher leakage volume at BBB dysfunction areas was shown to be associated with aggravation of lesion severity in the white matter 37,240,241.

Another contributor of WMLs during hypoperfused and inflammatory states is the redistribution of the water channel aquaporin-4 (AQP4). AQP4 is abundantly expressed on astrocytic end feet process, contributing to the polarity of astrocytic cells and BBB integrity 242,243. CCH-driven redistribution of AQP4 mediates osmotically-driven water transport into the brain parenchyma, resulting in edema within the white matter regions, and hence WML formation 244,245. This fluid accumulation appears as white matter hyperintensities on magnetic resonance imaging (MRI), which is one of the diagnostic criteria in identifying VaD patients.

## Clinical methods to detect BBB breakdown

**Figure 5:**
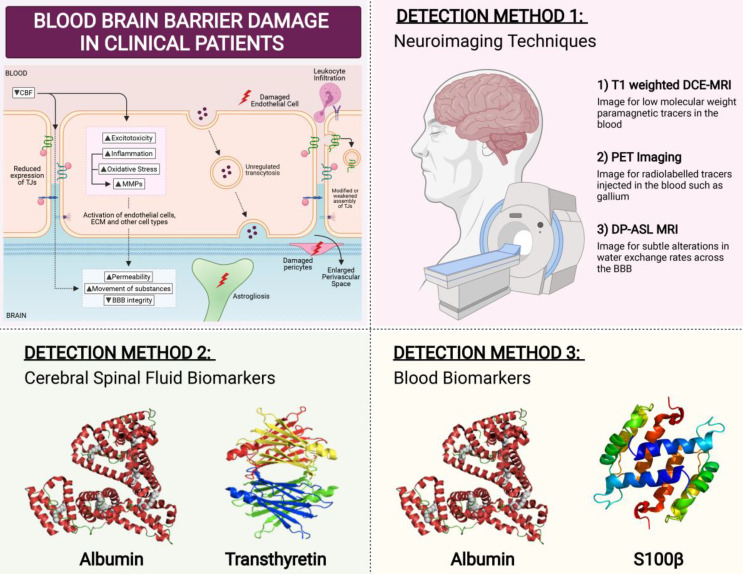
Clinical Detection Method for BBB Breakdown. In patients, there are three main ways that BBB breakdown is measured. The first detection method is via neuroimaging techniques. The most sensitive and commonly used method is T1-weighted dynamic contrast-enhanced MRI (DCE-MRI), to image for low molecular weight paramagnetic tracers in the blood. Positron emission tomography (PET) imaging visualises the movement of radiolabelled tracers injected such as gallium. However, these methods are quite invasive in nature. A non-invasive MRI method known as diffusion-prepared arterial spin labelling (DP-ASL) is an effective technique to measures subtle dysfunctions associated with altered water exchange rates across the BBB. Another detection method of BBB permeability is through the measurement of fluid biomarkers. While cerebral spinal fluid (CSF) albumin and transthyretin have been studied to be effective markers of BBB permeability, their invasive nature proves to be ineffective. Hence blood biomarkers Albumin and S100b have gained much interest lately in measuring BBB breakdown.

Several techniques can measure BBB permeability in humans **(Figure 5)**. The ratio of cerebrospinal fluid (CSF) and serum levels of plasma protein albumin correlates with BBB permeability. Serum albumin is usually not synthesized within the CNS, and so when detected in CSF fluid it is indicative of increased BBB permeability 246. Another method is serum analysis of CNS proteins such as serum S100β. S100β is not usually synthesised outside the CNS, hence its detection in the serum correlates with BBB permeability 247. Consistent with this phenomenon, elevated serum S100β levels have been reported in VaD patients 248. Similar to S100β, CSF protein transthyretin is extravasated when BBB is damaged 249. While transthyretin has not been studied in the context of VaD, CSF transthyretin levels have been implicated in other dementias with vascular contributions 250. However, biochemical assays involving CSF sampling is invasive which represents a major limitation for detection assays.

Brain imaging techniques can be used to assess the functional integrity of the BBB **(Figure 5)**. The most sensitive and commonly used method is T1-weighted dynamic contrast-enhanced MRI (DCE-MRI), to image for low molecular weight paramagnetic tracers in the blood 251. DCE-MRI has been employed to detect BBB breakdown in VaD patients 252. In preclinical models of vascular diseases, PET imaging of BBB breakdown has also been reported, albeit in limited numbers 253. PET imaging visualises the movement of radiolabelled tracers injected such as gallium 254. There are many disadvantages in using neuroimaging including its high cost, slow-processing time, and high invasiveness. However, perhaps the greatest limitation is that only advanced stages of BBB dysfunction can be detected, as neuroimaging is not sensitive enough to detect small changes in the BBB 255. In a continuum of disease progression such as occurs in VCI, it is important to identify BBB breakdown at early stages when BBB changes are relatively minor. This may be an important factor whereby BBB disruption could serve as a predictor for identification of at-risk dementia patients, so as to administer treatment options as early as possible.

A potentially effective measure of BBB dysfunction is through the use of a non-invasive MRI method known as diffusion-prepared arterial spin labelling (DP-ASL). This method measures subtle dysfunctions associated with altered water exchange rates across the BBB. Mapping the water exchange rate across the BBB without contrast imaging and invasiveness has been reported to have good reproducibility and has been proposed to be used as an imaging marker for cSVD and its associated cognitive impairment 256. The potential use of this method in clinics as a surrogate imaging biomarker for VCI and early dementia may help in identifying at-risk patients by modulating BBB damage early before the leakage of large molecules across the BBB occurs during more advanced stages of dementia **(Figure 5)**.

## Current progress in biomarker profiling of BBB dysfunction

Diagnostics spanning molecular biomarkers and neuroimaging techniques are important for indicating the presence of disease. Biomarkers specific for a particular disease would be ideal, yet they could also be broader in nature; for example, biomarkers for BBB dysfunction. One candidate for measuring BBB leakage is through radiolabelling of albumin protein and tracking its movement across the BBB. The invasive process of albumin tracing further limits its use in both clinical and research studies. Less invasive, yet effective biomarkers are necessary in the determination of BBB dysfunction. BBB dysfunction leads to parenchymal proteins moving into the circulation; therefore, parenchymal proteins may potentially be used as novel biomarkers to detect BBB dysfunction 257.

As our understanding of CCH improves, the ultimate goal of biomarker profiling for VCI and VaD will be to refine the biomarkers to deliver a disease-specific indicator. While these molecular markers have been correlated to BBB dysfunction, they have been reported to arise in various settings such as ischemic stroke, AD, traumatic brain injury and schizophrenia, rendering these markers unspecific to VCI 257-260. Increased BBB permeability is associated with increased brain pathology during CCH. Hence, there is a growing interest for potential therapeutic strategies to be introduced to target BBB damage and ultimately prevent the onset and progression of CCH-induced neuropathological features. Detailed understanding of the mediators of BBB disruption is key to finding potential treatments to prevent or reverse BBB breakdown in VaD. However a BBB dysfunction-related biomarker with specificity and sensitivity for VCI or VaD has not been reported to date. Prospective studies to evaluate the relationship between biomarkers of BBB dysfunction and VCI are likely to be an important area of study.

## Conclusion and perspectives

The BBB is an important barrier that maintains and regulates the brain microenvironment to allow for proper neuronal functioning. In the context of vascular cognitive disorders, the brain experiences a state of CCH, where the cells do not receive sufficient oxygen and nutrients. The brain cells then initiate a pathophysiological process through various mechanisms. At the BBB interface, abnormal changes in endothelial cells directly leads to impairment of the BBB, further reducing CBF in the brain. Damages to pericytes, astrocytes and surrounding microglia further aggravate CBF regulation, and induce further injury at the BBB. This review has elucidated the various known pathological drivers of BBB dysfunction and secondary injuries downstream. While there is indeed progress being made in the field regarding BBB dysfunction during CCH, many questions remain unanswered. For instance, which is the main pathway governing dynamic regulation of the BBB in response to CCH? Are there preferential areas of BBB breakdown occurring throughout the brain that account for differential impact of cognitive loss in humans? Research geared towards answering these questions is necessary to understand the mechanisms underlying CCH. Future studies in the field of VCI should target ways to reduce small vessel endothelial damage to prevent BBB damage and brain injury. These studies may include, improvement of lifestyle factors such as diet and exercise. Additional studies are needed to determine whether BBB dysfunction can predict 'at risk' white matter, and whether treatment targeting BBB dysfunction can reverse WML and cognitive impairment. Therapeutic interventions that promote the health of the BBB may provide opportunities for the brain to control the course of VCI development.
